# Supplementary material for: Multi‐dataset identification of innovative feature genes and molecular mechanisms in keratoconus
Source: J Cell Mol Med. 2024 Sep 19;28(18):e70079. doi: 10.1111/jcmm.70079 (PMC11412914; doi:10.1111/jcmm.70079)
Supplement: Supplementary file 2 — Material S2. [file JCMM-28-e70079-s002.zip › 7.GSEA analysis/1.ARL11/KEGG_SMALL_CELL_LUNG_CANCER.html]

Details for gene set KEGG\_SMALL\_CELL\_LUNG\_CANCER[GSEA]

|  || Dataset | ARL11.group.cls#h\_versus\_l |
| Phenotype | group.cls#h\_versus\_l |
| Upregulated in class | h |
| GeneSet | KEGG\_SMALL\_CELL\_LUNG\_CANCER |
| Enrichment Score (ES) | 0.21407671 |
| Normalized Enrichment Score (NES) | 0.8363601 |
| Nominal p-value | 0.65217394 |
| FDR q-value | 1.0 |
| FWER p-Value | 1.0 |
Table: GSEA Results Summary

  

Fig 1: Enrichment plot: KEGG\_SMALL\_CELL\_LUNG\_CANCER      
 Profile of the Running ES Score & Positions of GeneSet Members on the Rank Ordered List

  

| SYMBOL | TITLE | RANK IN GENE LIST | RANK METRIC SCORE | RUNNING ES | CORE ENRICHMENT || 1 | RARB | na | 584 | 0.459 | 0.0452 | Yes |
| 2 | TRAF5 | na | 1521 | 0.335 | 0.0636 | Yes |
| 3 | LAMB4 | na | 1566 | 0.332 | 0.1072 | Yes |
| 4 | PIK3R3 | na | 2180 | 0.284 | 0.1279 | Yes |
| 5 | FN1 | na | 2409 | 0.270 | 0.1579 | Yes |
| 6 | CCNE2 | na | 2488 | 0.266 | 0.1917 | Yes |
| 7 | PIK3CG | na | 2870 | 0.247 | 0.2141 | Yes |
| 8 | APAF1 | na | 5299 | 0.157 | 0.1653 | No |
| 9 | PIK3R1 | na | 6079 | 0.135 | 0.1611 | No |
| 10 | TRAF3 | na | 6905 | 0.114 | 0.1527 | No |
| 11 | COL4A1 | na | 7590 | 0.100 | 0.1465 | No |
| 12 | SKP2 | na | 8159 | 0.089 | 0.1422 | No |
| 13 | LAMA5 | na | 8273 | 0.087 | 0.1507 | No |
| 14 | IKBKB | na | 8698 | 0.079 | 0.1492 | No |
| 15 | PIK3R2 | na | 8783 | 0.078 | 0.1574 | No |
| 16 | AKT2 | na | 9348 | 0.070 | 0.1505 | No |
| 17 | TP53 | na | 9447 | 0.068 | 0.1569 | No |
| 18 | RXRA | na | 9938 | 0.061 | 0.1510 | No |
| 19 | PIK3R5 | na | 10118 | 0.059 | 0.1538 | No |
| 20 | MYC | na | 10444 | 0.054 | 0.1518 | No |
| 21 | TRAF2 | na | 11495 | 0.042 | 0.1271 | No |
| 22 | LAMB2 | na | 11967 | 0.036 | 0.1184 | No |
| 23 | MAX | na | 12403 | 0.032 | 0.1102 | No |
| 24 | CDKN2B | na | 13191 | 0.025 | 0.0909 | No |
| 25 | PIK3CA | na | 13512 | 0.023 | 0.0848 | No |
| 26 | XIAP | na | 14006 | 0.019 | 0.0731 | No |
| 27 | PTEN | na | 14202 | 0.017 | 0.0698 | No |
| 28 | BIRC2 | na | 14603 | 0.014 | 0.0602 | No |
| 29 | CCNE1 | na | 14811 | 0.013 | 0.0559 | No |
| 30 | CHUK | na | 15127 | 0.010 | 0.0482 | No |
| 31 | PIAS1 | na | 15768 | 0.005 | 0.0305 | No |
| 32 | RB1 | na | 16201 | 0.002 | 0.0183 | No |
| 33 | E2F2 | na | 16509 | 0.000 | 0.0095 | No |
| 34 | CCND1 | na | 16546 | -0.000 | 0.0084 | No |
| 35 | LAMB1 | na | 16555 | -0.000 | 0.0082 | No |
| 36 | CYCS | na | 17719 | -0.008 | -0.0242 | No |
| 37 | E2F1 | na | 17721 | -0.008 | -0.0231 | No |
| 38 | AKT1 | na | 18085 | -0.011 | -0.0321 | No |
| 39 | PIAS3 | na | 18227 | -0.012 | -0.0346 | No |
| 40 | CDK4 | na | 18688 | -0.015 | -0.0458 | No |
| 41 | ITGA2 | na | 19176 | -0.018 | -0.0575 | No |
| 42 | ITGA3 | na | 19278 | -0.019 | -0.0578 | No |
| 43 | ITGA2B | na | 19828 | -0.023 | -0.0706 | No |
| 44 | CDKN1B | na | 20317 | -0.026 | -0.0811 | No |
| 45 | CASP9 | na | 20358 | -0.026 | -0.0787 | No |
| 46 | FHIT | na | 20403 | -0.027 | -0.0764 | No |
| 47 | LAMA4 | na | 20981 | -0.031 | -0.0888 | No |
| 48 | TRAF6 | na | 21028 | -0.032 | -0.0859 | No |
| 49 | BIRC3 | na | 21919 | -0.039 | -0.1063 | No |
| 50 | PIAS2 | na | 22018 | -0.040 | -0.1037 | No |
| 51 | ITGA6 | na | 22097 | -0.041 | -0.1004 | No |
| 52 | RXRB | na | 22268 | -0.043 | -0.0996 | No |
| 53 | PTK2 | na | 22303 | -0.043 | -0.0948 | No |
| 54 | IKBKG | na | 22799 | -0.048 | -0.1026 | No |
| 55 | ITGAV | na | 23305 | -0.053 | -0.1099 | No |
| 56 | TRAF4 | na | 23331 | -0.054 | -0.1033 | No |
| 57 | LAMC1 | na | 23462 | -0.055 | -0.0996 | No |
| 58 | PIAS4 | na | 23627 | -0.058 | -0.0965 | No |
| 59 | PIK3CB | na | 23818 | -0.060 | -0.0939 | No |
| 60 | CKS1B | na | 23832 | -0.060 | -0.0861 | No |
| 61 | BCL2 | na | 24069 | -0.063 | -0.0844 | No |
| 62 | NFKBIA | na | 24454 | -0.069 | -0.0862 | No |
| 63 | TRAF1 | na | 24455 | -0.069 | -0.0769 | No |
| 64 | NOS2 | na | 24516 | -0.070 | -0.0692 | No |
| 65 | CDK6 | na | 24642 | -0.071 | -0.0632 | No |
| 66 | E2F3 | na | 25055 | -0.077 | -0.0647 | No |
| 67 | PIK3CD | na | 25759 | -0.087 | -0.0732 | No |
| 68 | COL4A2 | na | 25836 | -0.088 | -0.0636 | No |
| 69 | CDK2 | na | 25887 | -0.088 | -0.0531 | No |
| 70 | LAMC2 | na | 26469 | -0.098 | -0.0566 | No |
| 71 | PTGS2 | na | 26519 | -0.099 | -0.0446 | No |
| 72 | LAMB3 | na | 26727 | -0.103 | -0.0366 | No |
| 73 | LAMC3 | na | 26756 | -0.103 | -0.0235 | No |
| 74 | NFKB1 | na | 26767 | -0.104 | -0.0097 | No |
| 75 | COL4A4 | na | 26954 | -0.107 | -0.0006 | No |
| 76 | AKT3 | na | 27547 | -0.120 | -0.0015 | No |
| 77 | LAMA3 | na | 28015 | -0.131 | 0.0028 | No |
| 78 | ITGB1 | na | 28114 | -0.134 | 0.0182 | No |
| 79 | COL4A6 | na | 28477 | -0.143 | 0.0270 | No |
| 80 | BCL2L1 | na | 29160 | -0.161 | 0.0291 | No |
| 81 | RELA | na | 29186 | -0.162 | 0.0503 | No |
| 82 | LAMA2 | na | 31294 | -0.231 | 0.0208 | No |
| 83 | LAMA1 | na | 31863 | -0.255 | 0.0389 | No |
| 84 | RXRG | na | 33017 | -0.323 | 0.0494 | No |
Table: GSEA details [plain text format]

  

Fig 2: KEGG\_SMALL\_CELL\_LUNG\_CANCER      
 Blue-Pink O' Gram in the Space of the Analyzed GeneSet

  

Fig 3: KEGG\_SMALL\_CELL\_LUNG\_CANCER: Random ES distribution      
 Gene set null distribution of ES for **KEGG\_SMALL\_CELL\_LUNG\_CANCER**
